# Supplementary material for: Standard ingredient of Drosophila medium reduces transmission and virulence of the gut pathogen Pseudomonas entomophila
Source: Microbiol Spectr. 2025 Aug 12;13(9):e03065-24. doi: 10.1128/spectrum.03065-24 (PMC12403818; doi:10.1128/spectrum.03065-24)
Supplement: Supplemental material — Tables S1 to S4; Fig. S1 to S5. [file spectrum.03065-24-s0001.pdf]

# Standard ingredient of *Drosophila* medium reduces transmission and virulence of the gut pathogen *Pseudomonas entomophila*

Youn Henry, Berta Canal-Domènech, Jaime González, Christine La Mendola, Tadeusz J. Kawecki

## Supplementary material

Figure S1

Figure S2

Figure S3

Figure S4

Figure S5

Table S1

Table S2

Table S3

Table S4

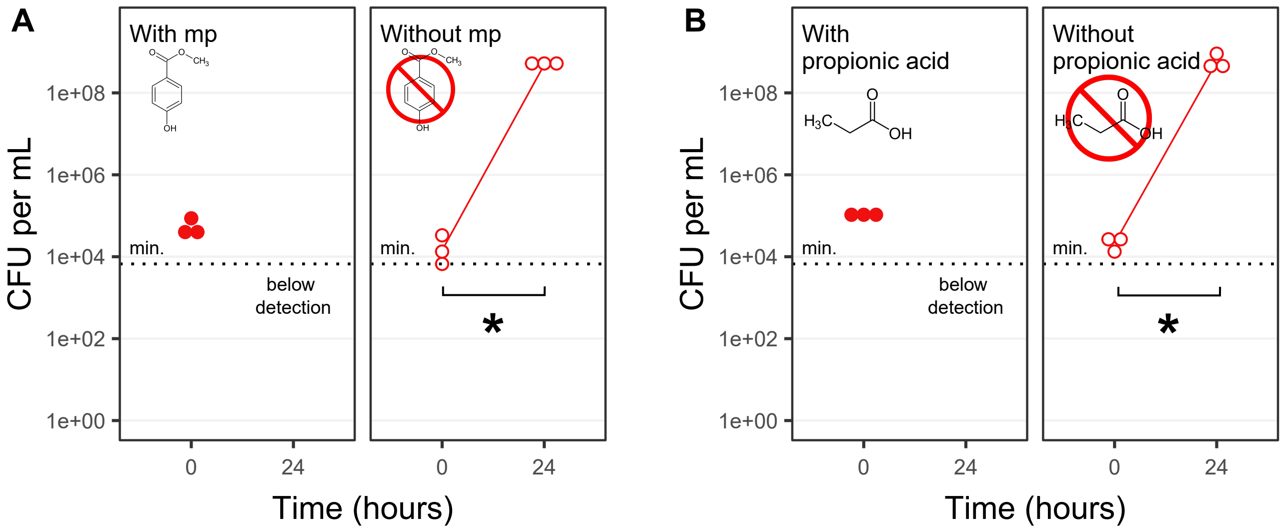
A
With mp
min.
below
detection
Without mp
min.
*
B
With
propionic acid
min.
below
detection
Without
propionic acid
min.
*
CFU per mL
1e+08
1e+06
1e+04
1e+02
1e+00
0
24
Time (hours)

Figure S1: *In vitro* growth of Pe in the presence or not of food preservative. In (A) we tested the effect of mp, and in (B) we tested the effect of propionic acid. Dots represent raw CFU measurements of N = 3 replicates. The solid dots with solid line are the condition with preservative, and the open dots with dashed line are the condition without preservative. The lower limit of detection was $6.6\times10^{3}$ CFU per mL (indicated with the horizontal dotted line), and the higher limit of detection was $1.0\times10^{10}$ CFU per mL.

Method:

We tested the bactericidal or bacteriostatic effect of methylparaben on Pe *in vitro,* at a concentration comparable to what is used in artificial fly diets. In a 96 wells microplate, we added 1% of ethanol or 1% of ethanol containing 20% methylparaben to a standardized suspension of Pe ($OD_{600}$ 0.0005) in LB medium, with 3 replicated wells per condition. We immediately plated a serial dilution of suspension from each well with PBS on *Pseudomonas* isolation agar to obtain estimates of initial bacterial concentration. We incubated the microplate at 28°C with agitation. After 24h of incubation, we plated another serial dilution with PBS from each well on *Pseudomonas* isolation agar. We counted the colonies after incubation of the plates for 20h at room temperature. We also tested the effect of propionic acid, another widely used antifungal additive, on Pe. With the same procedure and design as for mp, we compared growth of Pe exposed to 0.5% propionic acid or to 0.5% PBS buffer control.

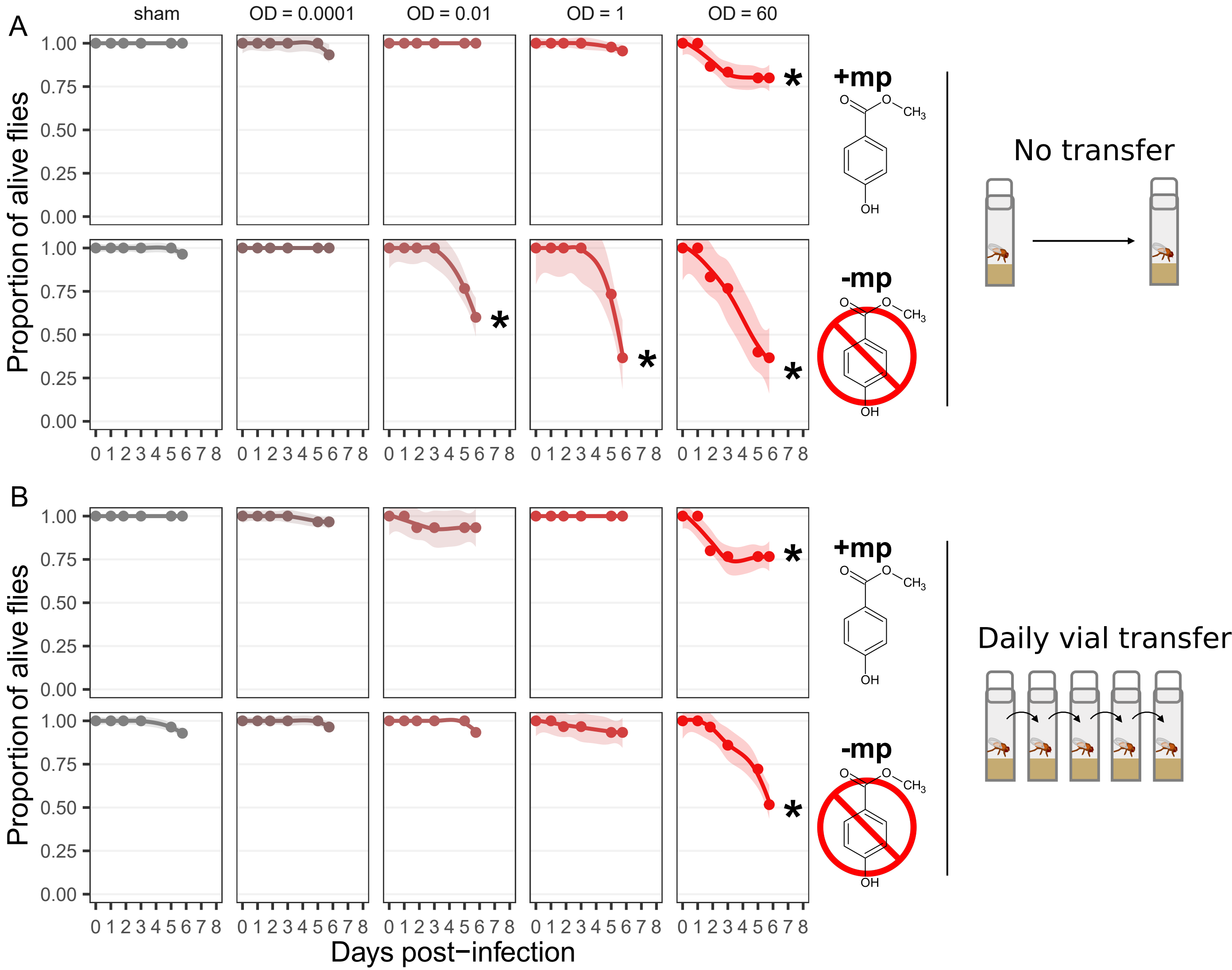

A
B
sham
OD = 0.0001
OD = 0.01
OD = 1
OD = 60
Proportion of alive flies
1.00
0.75
0.50
0.25
0.00
0 1 2 3 4 5 6 7 8
Days post−infection
*
+mp
-mp
O
O
$CH_3$
OH
No transfer
Daily vial transfer

Figure S2: Survival of female flies exposed to different doses of pathogen (in columns), and in the presence or absence of 0.2% mp in the diet (in rows), either without (A) or with (B) daily vial change. The color gradient represents the increasing Pe dose, from grey (sham infection) to red (highest infection dose of $OD_{600}$ 60). Each dot is the average survival proportion of N = 2 replicated vials, with 15 flies each. The line represents a loess regression on non-averaged proportions and the shaded ribbon the 95% confidence interval on this regression. The "*" indicates non-overlapping credible intervals on the last day of sampling from the posterior distribution, compared to the sham-infected condition. These results come from an independent replicate of the experiment presented in figures 2A and 3A, except for the maximum OD that was 60 instead of 50, and for the fly food which was not used as fresh, leaving time for commensal bacteria to potentially grow on the surface before the flies were added.

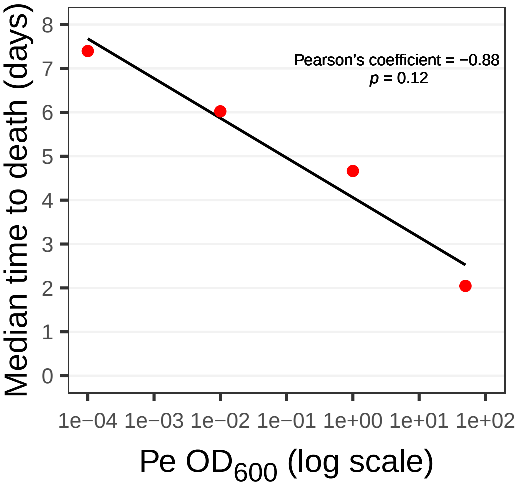
Median time to death (days)
8
7
6
5
4
3
2
1
0
Pearson's coefficient = −0.88
p = 0.12
1e−04
1e−03
1e−02
1e−01
1e+00
1e+01
1e+02
Pe OD600 (log scale)

Figure S3: Correlation between the median time to death and the Pe dose. Dots show the median time to death extracted from loess regressions of survival data (y axis) as a function of the inoculated Pe dose at the start of the experiment (x axis), only keeping the Pe-infected flies maintained on -mp diet. The black line is the linear regression on the dots.

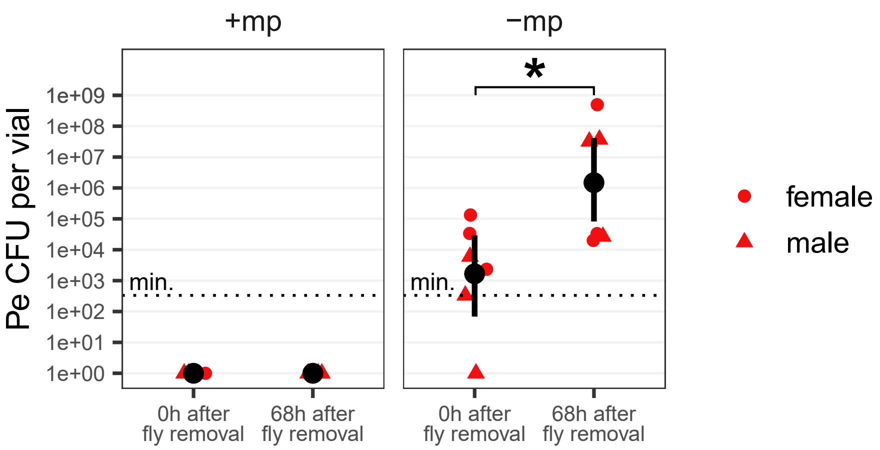
+mp
−mp
Pe CFU per vial
1e+09
1e+08
1e+07
1e+06
1e+05
1e+04
1e+03
1e+02
1e+01
1e+00
min.
min.
*
0h after fly removal
68h after fly removal
0h after fly removal
68h after fly removal
female
male

Figure S4: Environmental Pe load present on +mp or -mp fly food contaminated by Pe-infected flies. Groups of 5 males or 5 females were infected for 6h with Pe at OD 0.01 or with a sham infection. Groups were then transferred into new vials containing fresh food and were removed after 20h spent in those vials. We measured the Pe load right after removing the flies (t 0h) or after 3 additional days of incubation (t 68h). The sampling was destructive and each timepoint corresponds to different vials. Each red symbol is the CFU count of one vial plated on *Pseudomonas* isolation medium, with circles for vials contaminated by groups of females, and triangles for vials contaminated by groups of males. The black dots with error bars represent the average CFU per vial including both sexes, with 95% confidence intervals. Pairwise comparisons with “*” indicate non-overlapping credible intervals of the estimates from the posterior distribution. The sham control infection never showed colonies and therefore is not included in this representation.

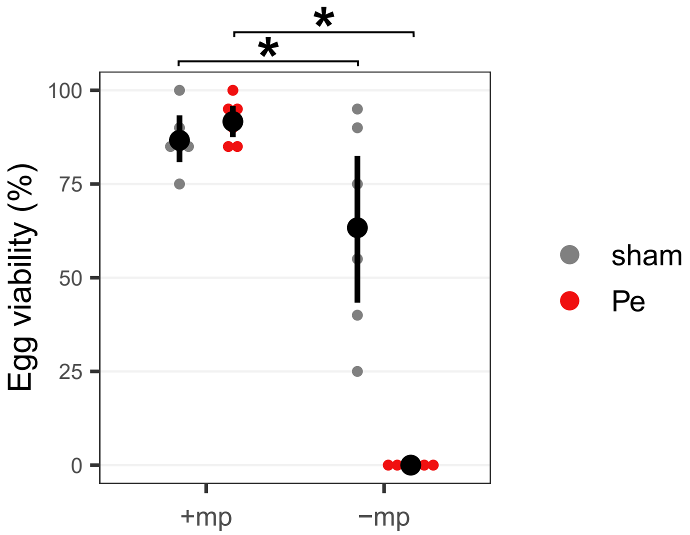

*
*
Egg viability (%)
100
75
50
25
0
+mp
−mp
sham
Pe

Figure S5: Pupation success of eggs developed on mp+ or mp- diet, contaminated for 24h by five adult males infected with Pe or with a sham infection. Pupation was recorded after six days, checking that no larvae remained in the medium. Each dot is the pupation success of a single vial containing 20 eggs at the start (N = 6 vials per condition). The black dots with error bars represent the average pupation success across replicates, with 95% confidence intervals. Pairwise comparisons with "*" indicate non-overlapping credible intervals of the estimates from the posterior distribution.

Table S1: Summary of the literature review. Only the articles using oral Pe infections are shown here. A dash indicates missing information.

| Article | Methylparaben use | Suspected methylparaben use | Propionic acid use | Pe optical density |
|---|---|---|---|---|
| Al Zouabi et al. (2023) | - | yes | - | 200 |
| Babin et al. (2014) | yes | yes | no | 100 |
| Beebe et al. (2015) | no | no | no | 0.01-10 |
| Beehler-Evans et al. (2015) | - | yes | - | 10-20 |
| Chakrabarti et al. (2012) | - | yes | - | 100 |
| Chakrabarti et al. (2014) | yes | yes | yes | 100 |
| Deshpande et al. (2022) | no | no | yes | 200 |
| Faria et al. (2015) | - | yes | - | 50 |
| Frochaux et al. (2020) | yes | yes | yes | 100 |
| Hegan et al. (2007) | no | no | no | 1 |
| Jacob et al. (2017) | - | yes | no | - |
| Joye et al. (2019) | yes | yes | no | 100 |
| Kawecki et al. (2020) | yes | yes | no | 100 |
| Kobler et al. (2020) | yes | yes | yes | 100 |
| Kuraishi et al. (2011) | - | yes | - | 100 |
| Liehl et al. (2006) | - | yes | - | 100 |
| Loch et al. (2017) | yes | yes | no | - |
| Martins et al. (2013) | - | yes | - | 50 |
| Onuma et al. (2023) | yes | yes | yes | 200 |
| Opota et al. (2011) | - | yes | - | 100 |
| Papagiannoulis et al. (2010) | - | yes | - | 200 |
| Prakash et al. (2023) | yes | yes | no | 45 |
| Sella et al. (2024) | - | yes | - | 100 |
| Sharda et al. (2022) | yes | yes | no | 300 |
| Shibata et al. (2015) | - | yes | - | 200 |
| Siva-Jothy et al. (2018) | yes | yes | no | 100 |
| Sleiman et al. (2015) | yes | yes | yes | 100 |
| Soory et al. (2022) | - | yes | - | 200 |
| Vallet-Gely et al. (2010a) | - | yes | - | 100 |
| Vallet-Gely et al. (2010b) | - | yes | - | 100 |
| Vijendravarma et al. (2015) | yes | yes | yes | 25 |
| Vodovar et al. (2006) | - | yes | - | 200 |
| Won et al. (2023) | - | yes | - | 200 |

## Methods

We performed a simple literature review, aiming to identify the proportion of published articles using antifungal preservatives in experiments involving Pe infection of fruit flies. In Web of Science, we used the keyword combination "*Drosophila*" + "*Pseudomonas entomophila*", searching in all fields. This approach excluded some rare articles only mentioning these terms in the main text, but we deemed our research sufficient to

provide a representative overview of the published record. We manually inspected the 64 initial hits and only kept articles explicitly using oral infection procedure, keeping a total of 33 articles. For all articles we collected information on the use of mp, the use of propionic acid, and the Pe $OD_{600}$ for infection. Because diet recipes were often omitted from the methods, we also recorded the "suspected use of mp". Articles with "suspected use of mp" contained no explicit information on mp use but were using some sort of "standard diet". We manually searched whether this "standard diet" contained mp in previous publications from the same research group, and if yes, we considered the article of interest had a "suspected use of mp".

## Results

We found a total of 64 hits for articles working on Drosophila infection with Pe. Out of these total hits, [33/64] articles performed oral infections, and [30/33] were likely maintaining flies on diet containing mp (down to [13/16] when only considering articles with explicit mention of the diet composition). All articles using mp performed infections with high Pe doses in the $OD_{600}$ range of 25-300. Out of the three articles with mp-free fly diet, one contained propionic acid and used $OD_{600}$ 200 for infections, and two others contained no preservative and used low infection doses ($OD_{600}$ 10, and $OD_{600}$ 1).

## Bibliography

Al Zouabi, L., Stefanutti, M., Roumeliotis, S., Le Meur, G., Boumard, B., Riddiford, N., Rubanova, N., Bohec, M., Gervais, L., Servant, N., et al. (2023). Molecular underpinnings and environmental drivers of loss of heterozygosity in Drosophila intestinal stem cells. CELL REPORTS 42,.

Babin, A., Kolly, S., Schneider, F., Dolivo, V., Zini, M. and Kawecki, T. (2014a). Fruit flies learn to avoid odours associated with virulent infection. BIOLOGY LETTERS 10,.

Beebe, K., Park, D., Taghert, P. and Micchelli, C. (2015). The Drosophila prosecretory transcription factor dimmed Is dynamically regulated in adult enteroendocrine cells and protects against gram-negative infection. G3-GENES GENOMES GENETICS 5, 1517–1524.

Beehler-Evans, R. and Micchelli, C. (2015). Generation of enteroendocrine cell diversity in midgut stem cell lineages. DEVELOPMENT 142, 654–664.

Chakrabarti, S., Liehl, P., Buchon, N. and Lemaitre, B. (2012). Infection-induced host translational blockage inhibits immune responses and epithelial renewal in the Drosophila gut. CELL HOST & MICROBE 12, 60–70.

Chakrabarti, S., Poidevin, M. and Lemaitre, B. (2014). The Drosophila MAPK p38c regulates oxidative stress and lipid homeostasis in the intestine. PLOS GENETICS 10,.

Deshpande, R., Lee, B. and Grewal, S. (2022). Enteric bacterial infection in Drosophila induces whole-body alterations in metabolic gene expression independently of the immune deficiency signaling pathway. G3-GENES GENOMES GENETICS 12,.

Faria, V., Martins, N., Paulo, T., Teixeira, L., Sucena, É. and Magalhaes, S. (2015). Evolution of Drosophila resistance against different pathogens and infection routes entails no detectable maintenance costs. EVOLUTION 69, 2799–2809.

Frochaux, M., Sleiman, M., Gardeux, V., Dainese, R., Hollis, B., Litovchenko, M., Braman, V., Andreani, T., Osman, D. and Deplancke, B. (2020). cis-regulatory variation modulates susceptibility to enteric infection in the Drosophila genetic reference panel. GENOME BIOLOGY 21,.

Hegan, P., Mermall, V., Tilney, L. and Mooseker, M. (2007). Roles for Drosophila melanogaster myosin IB in maintenance of Enterocyte brush-border structure and resistance to the bacterial pathogen Pseudomonas entomophila. MOLECULAR BIOLOGY OF THE CELL 18, 4625–4636.

Jacob, K., Rasmussen, A., Tyler, P., Servos, M., Sylla, M., Prado, C., Daniele, E., Sharp, J. and Purdy, A. (2017). Regulation of acetyl-CoA synthetase transcription by the CrbS/R two-component system is conserved in genetically diverse environmental pathogens. PLOS ONE 12,.

Joye, P. and Kawecki, T. (2019). Sexual selection favours good or bad genes for pathogen resistance depending on males' pathogen exposure. PROCEEDINGS OF THE ROYAL SOCIETY B-BIOLOGICAL SCIENCES 286,.

Kawecki, T. (2020). Sexual selection reveals a cost of pathogen resistance undetected in life-history assays. EVOLUTION 74, 338–348.

Kobler, J., Jimenez, F., Petcu, I. and Kadow, I. (2020). Immune receptor signaling and the mushroom body mediate post-ingestion pathogen avoidance. CURRENT BIOLOGY 30, 4693-+.

Kuraishi, T., Binggeli, O., Opota, O., Buchon, N. and Lemaitre, B. (2011). Genetic evidence for a protective role of the peritrophic matrix against intestinal bacterial infection in Drosophila melanogaster. PROCEEDINGS OF THE NATIONAL ACADEMY OF SCIENCES OF THE UNITED STATES OF AMERICA 108, 15966–15971.

Liehl, P., Blight, M., Vodovar, N., Boccard, F. and Lemaitre, B. (2006). Prevalence of local immune response against oral infection in a Drosophila/Pseudomonas infection model. PLOS PATHOGENS 2, 551–561.

Loch, G., Zinke, I., Mori, T., Carrera, P., Schroer, J., Takeyama, H. and Hoch, M. (2017). Antimicrobial peptides extend lifespan in Drosophila. PLOS ONE 12,.

Martins, N., Faria, V., Teixeira, L., Magalhaes, S. and Sucena, E. (2013). Host adaptation is contingent upon the infection route taken by pathogens. PLOS PATHOGENS 9,.

Onuma, T., Yamauchi, T., Kosakamoto, H., Kadoguchi, H., Kuraishi, T., Murakami, T., Mori, H., Miura, M. and Obata, F. (2023). Recognition of commensal bacterial peptidoglycans defines Drosophila gut homeostasis and lifespan. PLOS GENETICS 19,.

Opota, O., Vallet-Gély, I., Vincentelli, R., Kellenberger, C., Iacovache, I., Gonzalez, M., Roussel, A., van der Goot, F. and Lemaitre, B. (2011). Monalysin, a novel β-pore-forming toxin from the Drosophila pathogen Pseudomonas entomophila, contributes to host intestinal damage and lethality. PLOS PATHOGENS 7,.

Papagiannoulis, A., Mathiopoulos, K. and Mossialos, D. (2010). Molecular detection of the entomopathogenic bacterium Pseudomonas entomophila using PCR. LETTERS IN APPLIED MICROBIOLOGY 50, 241–245.

Prakash, A., Monteith, K., Bonnet, M. and Vale, P. (2023). Duox and Jak/Stat signalling influence disease tolerance in Drosophila during Pseudomonas entomophila infection. DEVELOPMENTAL AND COMPARATIVE IMMUNOLOGY 147,.

Sella, Y., Broderick, N., Stouffer, K., Mcewan, D., Ausubel, F., Casadevall, A. and Bergman, A. (2024). Preliminary evidence for chaotic signatures in host-microbe interactions. MSYSTEMS.

Sharda, S., Kawecki, T. and Hollis, B. (2022). Adaptation to a bacterial pathogen in Drosophila melanogaster is not aided by sexual selection. ECOLOGY AND EVOLUTION 12,.

Shibata, T., Maki, K., Hadano, J., Fujikawa, T., Kitazaki, K., Koshiba, T. and Kawabata, S. (2015). Crosslinking of a peritrophic matrix protein protects gut epithelia from bacterial exotoxins. PLoS Pathogens 11,.

Siva-Jothy, J., Prakash, A., Vasanthakrishnan, R., Monteith, K. and Vale, P. (2018). Oral bacterial infection and shedding in Drosophila melanogaster. JOVE-JOURNAL OF VISUALIZED EXPERIMENTS.

Sleiman, M., Osman, D., Massouras, A., Hoffmann, A., Lemaitre, B. and Deplancke, B. (2015). Genetic, molecular and physiological basis of variation in Drosophila gut immunocompetence. NATURE COMMUNICATIONS 6,.

Soory, A. and Ratnaparkhi, G. (2022). SUMOylation of Jun fine-tunes the Drosophila gut immune response. PLOS PATHOGENS 18,.

Vallet-Gely, I., Novikov, A., Augusto, L., Liehl, P., Bolbach, G., Péchy-Tarr, M., Cosson, P., Keel, C., Caroff, M. and Lemaitre, B. (2010a). Association of hemolytic activity of Pseudomonas entomophila, a versatile soil bacterium, with cyclic lipopeptide production. APPLIED AND ENVIRONMENTAL MICROBIOLOGY 76, 910–921.

Vallet-Gely, I., Opota, O., Boniface, A., Novikov, A. and Lemaitre, B. (2010b). A secondary metabolite acting as a signalling molecule controls Pseudomonas entomophila virulence. CELLULAR MICROBIOLOGY 12, 1666–1679.

Vijendravarma, R., Narasimha, S., Chakrabarti, S., Babin, A., Kolly, S., Lemaitre, B. and Kawecki, T. (2015). Gut physiology mediates a trade-off between adaptation to malnutrition and susceptibility to food-borne pathogens. ECOLOGY LETTERS 18, 1078–1086.

Vodovar, N., Vallenet, D., Cruveiller, S., Rouy, Z., Barbe, V., Acosta, C., Cattolico, L., Jubin, C., Lajus, A., Segurens, B., et al. (2006). Complete genome sequence of the entomopathogenic and metabolically versatile soil bacterium Pseudomonas entomophila. NATURE BIOTECHNOLOGY 24, 673–679.

Won, C., Nam, K., Ko, D., Kang, B. and Lee, I. (2023). NSD Overexpression in the fat body Increases antimicrobial peptide production by the immune deficiency pathway in Drosophila. INTERNATIONAL JOURNAL OF MOLECULAR SCIENCES 24,.

Table S2: Summary of model predictions for pairwise differences represented in figure 1. In the upper table, Δ are predicted for same treatment at 0 and 24h showing the time effect. In the lower table, Δ are predicted for same time with or without mp, showing the mp effect. We only interpret differences with confidence intervals not overlapping 0 as biologically meaningful and highlighted them in green or red for positive and negative differences respectively.

| Treatment | Time | Prediction | LowerCI | UpperCI |
|---|---|---|---|---|
| mp+ - mp- | 0 | 2.2e+04 | -1.1e+04 | 6.9e+04 |
| mp+ - mp- | 24 | **-3.9e+08** | -7.1e+08 | -8.7e+07 |

| Treatment | Time | Prediction | LowerCI | UpperCI |
|---|---|---|---|---|
| mp- | 24 - 0 | **3.9e+08** | 8.7e+07 | 7.1e+08 |
| mp+ | 24 - 0 | **-4.3e+04** | -8.0e+04 | -1.1e+04 |

Table S3: Summary of model predictions for pairwise survival differences represented in figures 2A and S2. The Δ are pairwise predicted survival differences at final time between sham infected and a given Pe infection dose, with same diet (+mp/-mp) and same vial change (with/without daily vial change). We only interpret differences with confidence intervals not overlapping 0 as biologically meaningful and highlighted them in green or red for positive and negative differences respectively.

| Pe dose | Mp | Vial change | Prediction | LowerCI | UpperCI |
|---|---|---|---|---|---|
| OD = 0.0001 - sham | mp- | no change | **-0.38** | -0.55 | -0.20 |
| OD = 0.01 - sham | mp- | no change | **-0.51** | -0.68 | -0.33 |
| OD = 1 - sham | mp- | no change | **-0.70** | -0.84 | -0.56 |
| OD = 50 - sham | mp- | no change | **-0.86** | -0.94 | -0.77 |
| OD = 0.0001 - sham | mp+ | no change | -0.04 | -0.14 | 0.04 |
| OD = 0.01 - sham | mp+ | no change | -0.01 | -0.09 | 0.06 |
| OD = 1 - sham | mp+ | no change | -0.06 | -0.16 | 0.03 |
| OD = 50 - sham | mp+ | no change | **-0.83** | -0.94 | -0.71 |
| OD = 0.0001 - sham | mp- | daily change | -0.05 | -0.12 | 0.01 |
| OD = 0.01 - sham | mp- | daily change | -0.06 | -0.14 | 0.01 |
| OD = 1 - sham | mp- | daily change | **-0.16** | -0.28 | -0.05 |
| OD = 50 - sham | mp- | daily change | **-0.94** | -0.99 | -0.88 |
| OD = 0.0001 - sham | mp+ | daily change | -0.03 | -0.10 | 0.02 |
| OD = 0.01 - sham | mp+ | daily change | 0.00 | -0.03 | 0.04 |
| OD = 1 - sham | mp+ | daily change | -0.03 | -0.10 | 0.02 |
| OD = 50 - sham | mp+ | daily change | **-0.94** | -0.99 | -0.88 |

Table S4: Summary of model predictions for pairwise Pe load differences represented in figures 2B and S2. In (A) the Δ are pairwise predicted Pe load differences at final time between sham infected and a given Pe infection dose, with same diet (+mp/-mp) and same vial change (with/without daily vial change). In (B) the Δ are pairwise predicted Pe load differences at final time between flies daily changed to new food or not, with same diet (+mp/-mp) and same Pe infection dose ($OD_{600}$ = 50, 1, 0.1, 0.0001, 0). We only interpret differences with confidence intervals not overlapping 0 as biologically meaningful and highlighted them in green or red for positive and negative differences respectively.

**A**

| Pe dose | Mp | Vial change | Prediction | LowerCI | UpperCI |
|---|---|---|---|---|---|
| OD = 0.0001 - sham | mp- | no change | **1.4e+05** | 3.6e+02 | 4.5e+05 |
| OD = 0.01 - sham | mp- | no change | **4.5e+05** | 1.1e+03 | 1.6e+06 |
| OD = 1 - sham | mp- | no change | **2.6e+05** | 7.4e+02 | 9.1e+05 |
| OD = 50 - sham | mp- | no change | **1.1e+06** | 3.8e+03 | 3.9e+06 |
| OD = 0.0001 - sham | mp+ | no change | 2.5e+00 | -3.3e+00 | 1.2e+01 |
| OD = 0.01 - sham | mp+ | no change | 3.1e+00 | -3.1e+00 | 1.4e+01 |
| OD = 1 - sham | mp+ | no change | 2.8e+00 | -3.4e+00 | 1.3e+01 |
| OD = 50 - sham | mp+ | no change | **4.2e+05** | 3.7e+02 | 1.5e+06 |
| OD = 0.0001 - sham | mp- | daily change | **5.8e+03** | 1.1e+01 | 2.0e+04 |
| OD = 0.01 - sham | mp- | daily change | **1.4e+03** | 8.8e-01 | 5.1e+03 |
| OD = 1 - sham | mp- | daily change | **1.9e+04** | 5.1e+01 | 6.7e+04 |
| OD = 50 - sham | mp- | daily change | **2.0e+05** | 4.9e+02 | 7.1e+05 |
| OD = 0.0001 - sham | mp+ | daily change | 7.5e+00 | -6.6e+00 | 3.3e+01 |
| OD = 0.01 - sham | mp+ | daily change | 4.7e+00 | -6.8e+00 | 2.3e+01 |
| OD = 1 - sham | mp+ | daily change | 3.1e+01 | -7.4e+00 | 1.2e+02 |
| OD = 50 - sham | mp+ | daily change | **1.7e+04** | 1.2e+01 | 5.9e+04 |

**B**

| Pe dose | Mp | Vial change | Prediction | LowerCI | UpperCI |
|---|---|---|---|---|---|
| sham | mp- | daily change - no change | -3.5e+01 | -1.2e+02 | 5.4e+00 |
| OD = 0.0001 | mp- | daily change - no change | -1.3e+05 | -4.7e+05 | 3.2e+04 |
| OD = 0.01 | mp- | daily change - no change | -4.5e+05 | -1.6e+06 | 7.4e+03 |
| OD = 1 | mp- | daily change - no change | -2.4e+05 | -9.3e+05 | 1.0e+05 |
| OD = 50 | mp- | daily change - no change | -9.2e+05 | -4.2e+06 | 1.0e+06 |
| sham | mp+ | daily change - no change | 6.0e-01 | -2.9e+00 | 4.8e+00 |
| OD = 0.0001 | mp+ | daily change - no change | 5.6e+00 | -1.5e+01 | 3.6e+01 |
| OD = 0.01 | mp+ | daily change - no change | 2.2e+00 | -1.9e+01 | 2.6e+01 |
| OD = 1 | mp+ | daily change - no change | 2.9e+01 | -2.3e+01 | 1.2e+02 |
| OD = 50 | mp+ | daily change - no change | -4.0e+05 | -1.5e+06 | 9.2e+04 |
